# Atopy and Lifestyle Survey of Allergic Patients From Urban Environment in Romania: Preliminary Data From an Interactive Qualifying Project

**DOI:** 10.7759/cureus.12714

**Published:** 2021-01-15

**Authors:** Polliana Mihaela Leru, Deanna Kay, Jessica Kelly, Tam Tuong, Tobias Schaeffer, Bland Addison, Rodica Neamtu

**Affiliations:** 1 Internal Medicine, Colentina Clinical Hospital/Carol Davila University of Medicine and Pharmacy, Bucharest, ROU; 2 Computer Science, Worcester Polytechnic Institute, Worcester, USA; 3 International Studies, Worcester Polytechnic Institute, Worcester, USA

**Keywords:** allergic patients, atopy, lifestyle survey, pollen allergies, urban environment

## Abstract

Introduction

Respiratory allergies represent an important public health problem, with increasing prevalence and severity in Europe during the last decades. The rise of pollen allergies is an issue that continues to negatively impact people’s daily lives across the globe and has become more important in the light of global warming and increasing air pollution. The aim of our paper is to evaluate the prevalence of declared atopy and the influence of lifestyle on allergic diseases, particularly on pollen allergies, in the urban environment from Romania.

Methods

The study is based on the cooperation Interactive Qualifying Project (IQP) called “Pollen Allergies in Romania: Optimizing Data Analysis in Raising Awareness”, agreed and carried on between a group of North-American students and teachers from Worcester Polytechnic Institute and a hospital-based allergy team from Carol Davila University of Medicine and Pharmacy and Colentina Clinical Hospital from Bucharest. The project aimed to evaluate the prevalence of atopy and lifestyle practices of allergic patients and to develop a data analysis tool to determine correlations between pollen counts and other environmental factors in the city of Bucharest.

Results

The lifestyle survey revealed that about one-third of allergic patients declared history of atopy. Some of the declared lifestyle practices can be considered environmental risk factors for allergies. This IQP can be considered a model of international, interdisciplinary and intercultural collaboration.

Conclusion

We concluded that Romania is facing an increasing pollen allergies trend and some actual lifestyle aspects can significantly influence the risk of pollen allergies in the big city environment.

## Introduction

The burden of allergic diseases is reported to be progressively increasing during the past five decades mostly in the developed industrialized countries [1]. Allergies are recognized as an important global epidemic, carrying a significant individual, social and economic burden [2].

Allergenic pollen is an important environmental risk factor for respiratory allergies, mostly for rhinitis and asthma and is negatively influenced by air pollution in an urban environment. Data from the literature also mentioned some lifestyle choices such as dietary restrictions, limited physical activity and early exposure to indoor pollutants as risk factors for developing an allergy [3].

Allergic rhinitis affects more than 20% of the population in European countries, including Romania, where local studies mentioned an increasing prevalence trend for more than ten years [4]. Allergic rhinitis and asthma are the most common manifestations of atopy, which is the genetic predisposition of some individuals to develop IgE-mediated hypersensitivity reactions to allergens.

Pollen allergies represent an issue with severe impact on people’s daily lives across the globe, being particularly heightened in Europe, where many invasive species thrive and produce allergenic pollen [5]. A growing interest and concern is due to the invasive ragweed - Ambrosia spp., which is rapidly spreading in many regions [6]. In Romania, pollen allergies have become significantly more frequent and severe in the past several years, as noticed by local studies from Bucharest [7,8]. Symptoms of pollen allergies may cause serious respiratory damage, so it is important to increase public awareness of the pollen levels in the atmosphere and provide resources to help prevent the consequent diseases.

The goals of our study, based on the Interactive Qualifying Project (IQP) called “Pollen Allergies in Romania: Optimizing Data Analysis in Raising Awareness”, were to evaluate the prevalence of atopy and relevant aspects referring to lifestyle of allergic patients in the urban environment of Bucharest, using a lifestyle survey, in order to raise public awareness of this important public health issue. We also aimed to find correlations between pollen count, chemical air pollutants and meteorological factors and to initiate a prospective analysis of pollen allergies by using the data analysis tool created by the Worcester Polytechnic Institute (WPI) team.

## Materials and methods

Our paper is based on the cooperation Interactive Qualifying Project (IQP) called “Pollen Allergies in Romania: Optimizing Data Analysis in Raising Awareness”, agreed and carried on between a group of North-American students and teachers from Worcester Polytechnic Institute, Massachusetts and a hospital-based allergy team from Carol Davila University of Medicine and Pharmacy and Colentina Clinical Hospital from Bucharest [9].

The project was remotely conducted online during March-April 2020, due to the outbreak of COVID-19 pandemic.

We developed and distributed a survey on lifestyle data, in order to demonstrate the prevalence of atopy and some of the Romanian activities which may impact allergy symptoms and pollen levels. The survey included questions directed to gather information about people’s background, self-reported history of atopy, their allergies (based on medical diagnosis), transportation usages, household conditions, possible occupational exposure to allergens and diet. The full survey can be found at https://docs.google.com/forms/d/e/1FAIpQLSdnq5O7HWmgkJXhcRB5Q3Z2cZRHjNijrUTMpqFiVhtPlOekXQ/viewform. The survey was refined in response to comments from our collaborators and advisors from the USA and Romania, as well as the responses of the participants. It was distributed to the Ambrosia sufferer Facebook group having 3000 participants and to other allergic patients from the allergist hospital records of the last three years. The public interest in allergy-related search terms over time was explored through Google Trends data available from Romania and further correlated with pollen and environmental data.

The survey was posted on the Facebook page of allergy sufferers group for maximum of three weeks, due to the short duration of the project, therefore we received 92 responses, representing a response rate of 3.06% only.

## Results

Characteristics of the responders group

The age of participants ranged from 18 to 74 years, with mean age 34 years, 74% being female. Referring to living area, 47% of respondents were from Muntenia region where Bucharest is located and 63% reported to grow up in urban areas. More than a quarter (28.2%) of the responders had more than one type of allergies. Eighty-seven percent of responders were affected by pollen allergies (including ragweed, trees, and grasses), 28% were affected by dust mites and the rest were affected by other allergens such as animals, chemicals, food, metals or declared having histamine intolerance. Sneezing, ocular itching, and nasal conditions such as runny nose, nasal congestion, and itchy nose were the most common symptoms, with a few sufferers experiencing rashes or hives.

History of atopy

**Figure 1:**
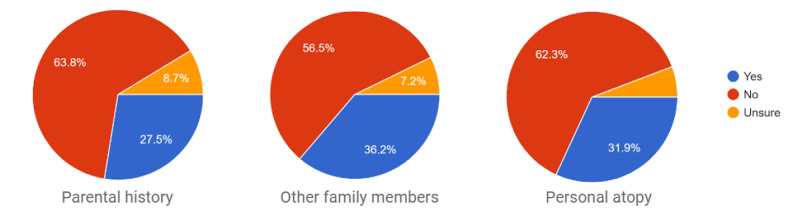
Declared history of atopy

Between 27.5% and 36.2% of the responders declared parental or other family members’ history of atopic diseases, mainly allergic rhinitis and asthma. We recorded that 31.9% of the responders had allergies in their early life or developed their allergies within their lifetimes which suggest personal history of atopy. A total of 68.5% responders have been suffering from their symptoms for over three years, up to the survey time (Figure 1).

Relevant aspects referring to lifestyle of allergic patients

**Figure 2:**
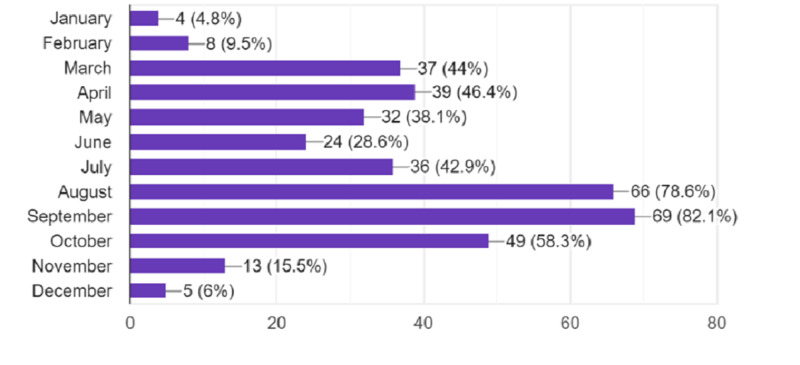
Monthly reported allergy symptoms

Many of the respondents reported to have allergy symptoms throughout the year, with 78.6% and 82.1% during August and September, which is the peak of Ambrosia season. More than 40% of people reported having their symptoms also during springtime, possibly suggesting that many seasonal pollens are involved (Figure 2).

Referring to the treatment of allergies, more than 65% of responders declared to ask doctor’s advise and 30.3% used non-prescription medication, while 35% waited for the symptoms to go away or used natural remedies.

**Figure 3:**
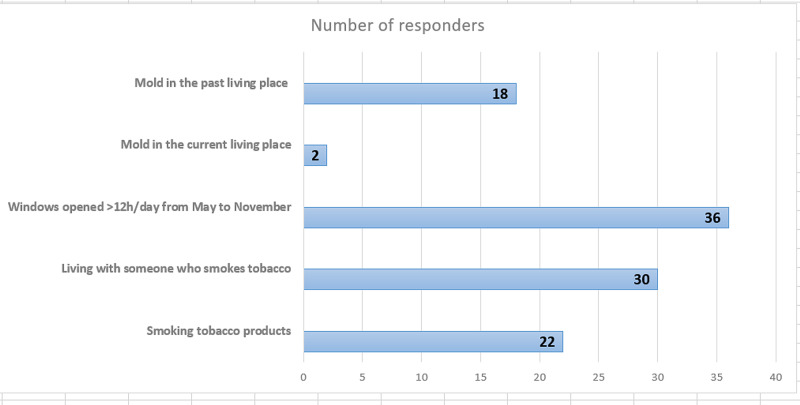
Smoking and living conditions

Referring to other environmental exposure to respiratory noxious substances, the survey revealed that 22 (30%) allergy sufferers reported that they smoke tobacco products and 30 (41%) were passive smokers. We also noted that 18 patients (25%) had lived in a space with mold and 36 (49%) used to keep their room windows opened more than 12 hours daily between May and November (Figure 3).

**Figure 4:**
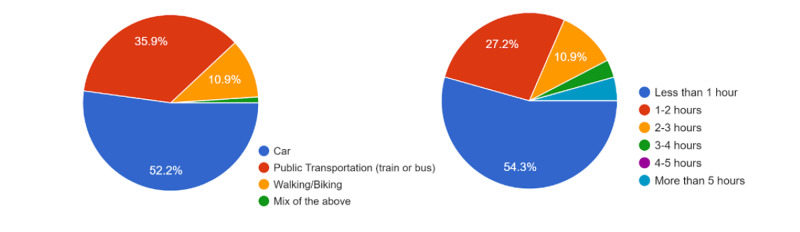
Primary transportation mode

Referring to the transportation mode, we found that 52% of Romanian responders chose personal cars as their primary mode of transportation and 54% declared to spend less than 1 hour driving per day (Figure 4).

Referring to dietary aspects, 90% of the allergy sufferers reported not having a restricted diet, with food choices from all categories, corresponding to food diversity. We also noted that 52% of the responders have or used to have pets.

An interesting finding is that 74.7% of Romanians declared to experience their symptoms at work which, considering the consequences of allergies, can affect their productivity and the quality of their work.

We also searched for the public interest in allergic diseases and we found from Google Trends that the terms “Ragweed” and “Allergy” in Romania became increasingly popular over the past five years, being higher during the season of pollen allergies [10].

## Discussion

This paper presents the preliminary results of the lifestyle survey and we shall continue to deploy a lifestyle survey geared towards allergic patients, in order to collect information detailing the lifestyle practices of pollen allergy patients from various regions of Romania. We discuss here our findings so far, while the newly collected data will be the main topic of another paper, as well as the correlation data.

As far as we know, this study is the first evaluation of the prevalence of atopy and lifestyle in allergic persons from Romania. Data from the literature mention an estimated prevalence of atopy in developed countries between 10-30% of the general population and about 80% of atopic persons have a family history of allergy, compared with 20% of non-atopic individuals [11]. Many authors consider that pollen allergies are not so much genetically driven and instead are more likely to be impacted by environmental factors. Early sensitization to environmental factors is considered to play a major role in developing some allergies, irrespective of atopy [12]. Due to the fact that sensitization to Ambrosia pollen seems to be not so much related to atopy, compared to other types of allergens and the majority of the respondents to our survey showed symptoms during the peak season of Ambrosia, the interpretation of declared atopy in this context might be a biased result.

There is still a large gap in our knowledge regarding global trends across different biogeographical regions and for a wider diversity of allergenic pollen [1]. As pollen allergies are rising in Romania, allergists are noticing the need to refine and develop local research in this field [7]. Data from the literature outline that the rise in pollen count and seasonal allergies are affected by city upkeep, climate change, and people’s lifestyle practices [13]. Statistics show that the number of patients reported from urban areas triples, sometimes quadruples, those from rural areas [14]. Cities provide an advantageous environment for weeds, which thrive uncontested in loosely packed soil and warmer temperatures.

One study showed that association of seasonal conjunctivitis to allergic rhinitis due to ragweed pollen was more frequent in patients from the urban area, compared with those from rural ones, possibly due to increased urban pollution [15].

Lifestyle is a complex topic that covers activities that directly and indirectly affect the chances of allergy development. Living in a congested city with exposure to high pollen concentration increases the chances of having an allergy [16]. By choosing cars as their primary mode of transportation, people are adding to ambient pollutants that are harmful to the respiratory system. Although it is true that the majority of Romanians choose cars as their primary mode of transportation, more studies are needed to determine the relationship between Romanian’s car usage and their allergies [17].

Other factors such as poor diet, hygiene, household conditions and limited exercise are related to a person’s overall health, adding to the negative effects of pollen allergies. For allergy patients, self-medication is common, due to underestimating the severity of allergies, and inaccessibility to proper healthcare [18].

Bucharest is a highly congested city with abundant air pollutants that are harmful to allergy sufferers [19]. The suburbs directly surrounding cities are most affected due to their combination of unmaintained growing space, urban pollution, and higher amount of plant life than the densely populated city [20]. This is further evidenced by Google Trends data, where ragweed was searched in Ilfov county, which surrounds Bucharest, more than anywhere else in Romania in the past five years.

The majority (75%) of the respondents reported experiencing their symptoms at work, which can affect their productivity, the quality of their work, and sometimes even their own safety.

Additional data should be collected over time to confirm the correlations between pollen and other environmental factors and to better understand Romanian lifestyle choices [21]. The possible interaction between viruses and pollen allergies, mainly due to COVID-19 pandemic, looks like a very useful research topic in the future [22].

Originality of our study

Our study is based on the particular Interactive Qualifying Project from WPI, which has been completely innovative and challenging for both American and Romanian teams. The project has been carried out remotely, in an intensive way, due to COVID-19 outbreak in March 2020. It can be considered a model of international, interdisciplinary and intercultural collaboration. The results reflect both the global interest of the topic of pollen allergies, the optimal interaction between two different medical and university systems and the real intention to bring relevant progress to the Romanian research in the field of allergic diseases.

Limitations of our study

The main limitation of our study is the short duration of the project and the reduced number of responders to the lifestyle survey, due to the pre-season and the pandemic period.

Since the survey was addressed mostly to Ambrosia sufferers, the results were somewhat biased towards those types of respondents. Therefore, more studies are necessary to have results that are representative for a larger population, especially for different subgroups of allergy sufferers.

## Conclusions

The results of our study have proved that Romania is observing a similar trend to other countries that are heavily impacted by allergenic pollen. The rate of declared atopy about 30% is close to that mentioned in the literature. Many of our lifestyle survey respondents were from urban areas, experienced allergy symptoms mostly during the Ambrosia season and experienced allergy symptoms while at workplace. Smoking and some living conditions can be considered environmental risk factors for respiratory allergies. More development of the pollen collection program is needed to predict and publicize daily pollen forecasts in the future. Further longer and larger studies are needed for a clear evaluation of the burden and risk factors of respiratory allergies in Romania.

## References

[1] Damialis A, Traidl-Hoffmann C, Treudler R (2019). Climate change and pollen allergies. In: Biodiversity and Health in the Face of Climate Change.

[2] Damialis A, Haring F, Gokkaya M (2019). Human exposure to airborne pollen and relationships with symptoms and immune responses: indoors versus outdoors, circadian patterns and meteorological effects in alpine and urban environments. Sci Total Environ.

[3] Gilles S, Akdis C, Lauener R, Schmid‐Grendelmeier P, Bieber T, Schäppi G, Traidl‐Hoffmann C (2018). The role of environmental factors in allergy: a critical reappraisal. Exp Dermatol.

[4] Agache I, Doros IC, Leru P, Bucur I, Poenaru M, Sarafoleanu C (2018). MP-AzeFlu provides rapid and effective allergic rhinitis control: results of a non-interventional study in Romania. Rhinology.

[5] Schmidt CW (2016). Pollen overload: seasonal allergies in a changing climate. Environ Health Perspect.

[6] Matyasovszky I, Makra L, Tusnády G (2018). Biogeographical drivers of ragweed pollen concentrations in Europe. Theor Appl Climatol.

[7] Leru PM, Eftimie AM, Thibaudon M (2018). First allergenic pollen monitoring in Bucharest and results of three years collaboration with European aerobiology specialists. Romanian J Intern Med.

[8] Leru PM, Eftimie AM, Anton VF, Thibaudon M (2019). Five-year data on pollen monitoring, distribution and health impact of allergenic plants in Bucharest and the southeastern region of Romania. Medicina.

[9] Worcester Polytechnic Institute (WPI (2021). Worcester Polytechnic Institute (WPI). Global projects program. http://www.wpi.edu/+globalprojects.

[10] (2021). Google Trends. Compare: allergy and ragweed. https://trends.google.com/trends/explore?date=today%205-y&geo=RO&q=%2Fm%2F0fd23,%2Fm%2F01yt1v.

[11] Blumenthal MN (2005). The role of genetics in the development of asthma and atopy. Curr Opin Allergy Clin Immunol.

[12] Erbas B, Lowe AJ, Lodge CJ (2013). Persistent pollen exposure during infancy is associated with increased risk of subsequent childhood asthma and hayfever. Clin Exp Allergy.

[13] D’Amato G, Cecchi L, D’Amato M, Liccardi G (2010). Urban air pollution and climate change as environmental risk factors of respiratory allergy: an update. J Investig Allergol Clin Immunol.

[14] Nicolaou N, Siddique N, Custovic A (2005). Allergic disease in urban and rural populations: increasing prevalence with increasing urbanization. Allergy.

[15] Majkowska-Wojciechowska B, Pełka J, Korzon L (2007). Prevalence of allergy, patterns of allergic sensitization and allergy risk factors in rural and urban children. Allergy.

[16] Abramson S (2018). Reducing environmental allergic triggers: policy issues. J Allergy Clin Immunol Pract.

[17] Te Q, Lianghua C (2020). Carsharing: mitigation strategy for transport-related carbon footprint. Mitig Adapt Strateg Glob Change.

[18] Manole M, Duma O, Gheorma A (2017). Self-medication - a public health problem in Romania nowadays. The first quest. Med Surg J.

[19] Gherasim C (2021). Green campaigners fume over Bucharest's pollution problem. https://www.euronews.com/2020/03/12/green-campaigners-fume-over-bucharest-s-pollution-problem.

[20] Cariñanos P, Casares-Porcel M (2011). Urban green zones and related pollen allergy: a review. Some guidelines for designing spaces with low allergy impact. Landscape Urban Planning.

[21] Medek DE, Simunovic M, Erbas B (2019). Enabling self-management of pollen allergies: a pre-season questionnaire evaluating the perceived benefit of providing local pollen information. Aerobiologia.

[22] (2021). Increased air pollution linked to far higher Covid-19 spread, study finds. https://medium.com/@airlyeu/increased-air-pollution-linked-to-far-higher-covid-19-spread-study-finds-fbd285682c92.

